# Polymer-Mediated Cryopreservation of Bacteriophages

**DOI:** 10.1021/acs.biomac.1c01187

**Published:** 2021-11-30

**Authors:** Huba L. Marton, Kathryn M. Styles, Peter Kilbride, Antonia P. Sagona, Matthew I. Gibson

**Affiliations:** † Department of Chemistry, University of Warwick, Coventry CV4 7AL, U.K.; ‡ Warwick Medical School, University of Warwick, Coventry CV4 7AL, U.K.; § School of Life Sciences, University of Warwick, Coventry CV4 7AL, U.K.; ∥ Asymptote, Cytiva, Chivers Way, Cambridge CB24 9BZ, U.K.

## Abstract

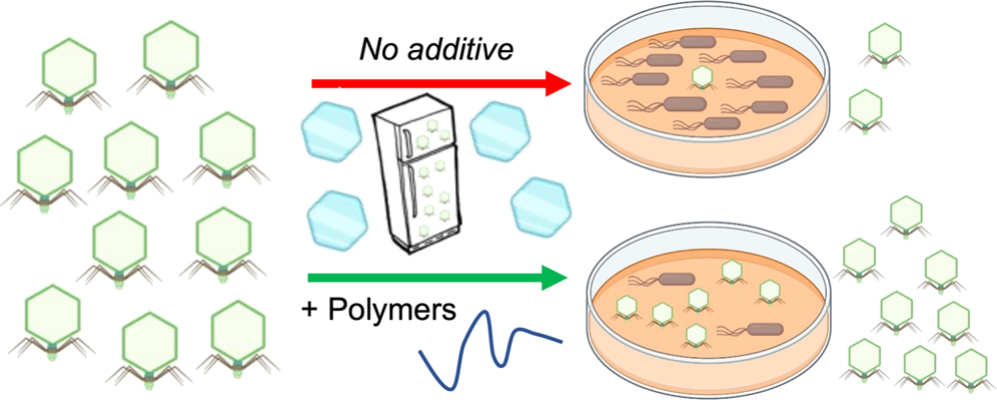

Bacteriophages (phages, bacteria-specific viruses) have biotechnological and therapeutic potential. To apply phages as pure or heterogeneous mixtures, it is essential to have a robust mechanism for transport and storage, with different phages having very different stability profiles across storage conditions. For many biologics, cryopreservation is employed for long-term storage and cryoprotectants are essential to mitigate cold-induced damage. Here, we report that poly(ethylene glycol) can be used to protect phages from cold damage, functioning at just 10 mg·mL^–1^ (∼1 wt %) and outperforms glycerol in many cases, which is a currently used cryoprotectant. Protection is afforded at both −20 and −80 °C, the two most common temperatures for frozen storage in laboratory settings. Crucially, the concentration of the polymer required leads to frozen solutions at −20 °C, unlike 50% glycerol (which results in liquid solutions). Post-thaw recoveries close to 100% plaque-forming units were achieved even after 2 weeks of storage with this method and kill assays against their bacterial host confirmed the lytic function of the phages. Initial experiments with other hydrophilic polymers also showed cryoprotection, but at this stage, the exact mechanism of this protection cannot be concluded but does show that water-soluble polymers offer an alternative tool for phage storage. Ice recrystallization inhibiting polymers (poly(vinyl alcohol)) were found to provide no additional protection, in contrast to their ability to protect proteins and microorganisms which are damaged by recrystallization. PEG’s low cost, solubility, well-established low toxicity/immunogenicity, and that it is fit for human consumption at the concentrations used make it ideal to help translate new approaches for phage therapy.

## Introduction

The use of biologic therapies (e.g., cells, proteins, viruses, vaccines) is rapidly growing, but there remain challenges to delivering them intact and functional to a patient.^1−5^ Bacteriophages (literally “bacteria eater”) or phages are viruses that specifically target and infect bacteria and are the most abundant organisms on earth.^6^ Competition between these viral predators and their bacterial hosts plays an important role in the evolutionary adaptations and diversification seen in many bacteria today.^7,8^ Generally, phages can be divided into virulent and temperate phages, the former carrying out a lytic replication cycle, where the phage uses the bacterial host to replicate by seizing the host’s molecular machinery and then escaping the cell to find a fresh host, the latter integrating into and then remaining dormant in the host genome as a “prophage” and replicating with the host genome in a lysogenic cycle.^9^ Phages are ubiquitous, from the depth of the oceans to hospital effluents.^10^ It is also becoming increasingly clear that phages play a role in the gut microbiome of the human body.^11,12^ In aquacultures (the farming of seafood), lytic phages have been used to alleviate pathogenic bacteria of a range of fish and shellfish.^13^ Phages have been approved for use as a food additive in meat products to protect consumers against *Listeria monocytogenes* by the Food and Drug Agency (FDA).^14^ Another use of lytic phages is to treat bacterial infections inside the human body (phage therapy).^10^ One of the positive attributes of phage therapy is that they can largely be applied without disruptions to the gut microbiota.^15^ The vast abundance of the phage in nature^16^ also means that there is almost an endless pipeline and so phages can be applied as “cocktails”, thereby reducing the chances of resistance developing to individual treatment.^17−19^

Phase II clinical trials on the bacteriophage are being undertaken, including against multidrug-resistant bacteria. For example, clinical improvement or full recovery was reported in up to 40% of 157 patients at the European Phage Therapy Unit (PTU) between 2008 and 2010;^20^ venous leg ulcers, using Intralytix phage cocktail WPP-201 targeting *Escherichia coli*,*Staphylococcus aureus*, and *Pseudomonas aeruginosa* reported no adverse effects;^21^ T4 coliphage cocktail and Microgen ColiProteus phage cocktail targeting *E. coli*, which reported no adverse effects, but the trial was terminated due to a lack of therapeutic effects.^22^ While the above are promising, no phage therapy has reached Phase III trials (to the best of our knowledge) nor is used as mainstream treatments in the USA or EU.^23^ This can partially be ascribed to a discrepancy between *in vitro* and *in vivo* data and a lack of understanding of the complex relationship between phages, bacteria, and human host^24−26^ but is also due to regulatory and commercial production and translation barriers. For example, there have been safety concerns relating to bacteriophage production for commercial use,^27^ with fears that the phage could transfer virulence factors from the bacteriophage’s host bacterium to pathogens in the patient.^28^ Problems with the commercial scale-up of bacteriophage purification were highlighted in the PhagoBurn phase I/II clinical trials, where work was halted multiple times, because of technological difficulties in bacteriophage production^29^ and regulatory barriers.

One important factor to consider when producing a commercially viable treatment is its storage options and stability over time (shelf-life). The biologic storage challenge has been highlighted during the development of vaccines for COVID-19, with several requiring sub −20 °C temperatures and hence appropriate cold-chain infrastructure to enable global roll-out.^3^ One reliable method for phage cryopreservation is storage inside the bacterial host.^30^ From a phage therapy point of view, however, the use of the infective phages requires the removal of the hosts, e.g., using chloroform and vigorous vortexing steps, and this comes with the concern that phage preparations are not always purified from their host endotoxins or potentially toxic purification reagents.^10,31^

Phage storage at ambient temperature is possible, but the success and longevity of this vary from phage to phage. For example, *Acinetobacter baumannii* phage vPhT2 was reported to have excellent stability in lysogeny broth but not in SM-II (a standard buffer for phage storage).^18^ Finding a suitable method for long-term storage for purified phages or developing preparations for standardized phage transport, storage, and use at the bed-side is important for their wider adoption. For example, many cell-based therapies are stored cryopreserved and thawed before use.^32,33^ Predictable cryopreservation outcomes are essential to control dosage, and in the case of cocktails, the thawed composition matches the frozen.

The cryopreservation of nucleated cells, bacteria, and proteins is typically achieved by the addition of (one or more) cryoprotectants to mitigate cold-induced damage, with dimethyl sulfoxide (DMSO) and glycerol being the most widely used cryoprotectants,^34−38^ but there is a desire to reduce or remove the volume of these used to increase post-thaw recovery and to reduce potential toxicity.^39−41^ Extremophiles survive in subzero climates by a series of adaptive mechanisms, which include the production of cryoprotectants, such as trehalose, glycerol, and osmolytes,^42−44^ as well as ice binding proteins (IBPs), which can prevent or promote ice formation and growth.^45^ There has been significant interest in developing synthetic materials to mimic the function of IBPs and other cryoprotectant molecules, with particular focus on their application in cryopreservation.^46−49^ Polyampholytes have been shown to be potent mammalian cell cryopreservation enhancers.^50−52^ Ice recrystallization inhibitors (IRIs) have also found application, with antifreeze proteins^53^ being shown to reduce hemolysis in erythrocyte cryopreservation and since have been studied in several cryopreservation scenarios.^54−56^ Bacteria and protein storage have been enhanced by IRIs,^57,58^ by preventing irreversible aggregation. The exact mechanism of protection (and damage) when using macromolecular cryoprotectants is still being studied and there is a need to compare how these materials can protect different organisms.

Here, we explore the use of synthetic polymers as low-concentration cryoprotectants for the bacteriophage of potential medical importance. Polymeric recrystallization inhibitors were tested but found not to provide additional protection to the phage during freeze/thaw. In contrast, the addition of (IRI inactive) poly(ethylene glycol), PEG, was found to enhance post-thaw recovery, in many cases allowing full recovery of phage at just 1 wt %, comparable to the positive control of 50 wt % glycerol (a known cryoprotectant). The bactericidal effect of the phage was also demonstrated to be retained post-thaw and initial experiments suggest that a range of water-soluble polymers could have this function, not just PEG. These observations show that macromolecular cryoprotectants for the phage can be formulated from simple off-the-shelf polymers and in particular may help develop frozen formulations for future phage-based therapies.

## Experimental Section

### Materials and Methods

Agarose, lysogeny broth (LB), poly(ethylene glycol) PEG (Mn 4000), poly(vinyl alcohol) (PVA) (MW 10 000, dialyzed), and poly(vinylpyrrolidone) (PVP) (Mn 40 000) were purchased from Sigma-Aldrich (Merck). Cesium chloride, magnesium sulfate heptahydrate, sodium chloride, and PEG (Mn 8000) were purchased from Fisher Scientific. Glycerol was purchased from Scientific Laboratory Supplies (SLS). Hydroxyethyl starch (HES) was purchased from Carbosynth. Phosphate-buffered solution (PBS) (8 g·L^–1^ NaCl, 0.2 g·L^–1^ KCl, 1.15 g·L^–1^ Na_2_HPO_4_, 0.2 g·L^–1^ KH_2_PO_4_) and Tris-HCl (24.2 g·L^–1^ Tris, 80 g·L^–1^ NaCl) were prepared by media preparation facility in the School of Life Sciences at the University of Warwick. SM-I buffer (1 M NaCl, 8 mM MgSO_4_·7H_2_O, 22.5 mM Tris-HCl pH 7.5) and SM-II buffer (100 mM NaCl, 8 mM MgSO_4_·7H_2_O, 22.5 mM Tris-HCl pH 7.5) was prepared in-house.

### Physical and Analytical Methods

#### Ice Recrystallization Inhibition Splat Assay

The ice recrystallization inhibition (IRI) activity of the PEG and PVA polymers was measured using a modified splat assay.^59^ A 10 μL sample of each polymer dissolved in SM buffer II was dropped 1.4 m onto a chilled glass coverslip placed on an aluminum plate on dry ice. Upon hitting the chilled coverslip, an ice wafer was formed instantaneously. The glass coverslip was transferred to a Linkam THMS600 cryostage and left to anneal at −8 °C under a N_2_ atmosphere for 30 min after taking an initial photograph at *t* = 0. Photographs (initial and after 30 min of annealing) were collected using an Olympus CX 41 microscope with a UIS-2 20×/0.45/∞/0-2/FN22 lens and crossed polarizers (Olympus Ltd., Southend-on-Sea, U.K.), which was equipped with a Canon DSLR 500D digital camera. Processing of each image was conducted using the freely available Fiji (ImageJ) software.^60^ In summary, the number of crystals in the 20× magnified images of the wafers were counted. Average values obtained were compared to the values of the SM-II buffer controls.

### Biological Methods

#### Viral Enrichment: Propagation of K1F-GFP and T4 Bacteriophages

To propagate the bacteriophage isolates, *E. coli* EV36 and *E. coli* AB1157 hosts for the K1F-GFP and T4 phage, respectively, were grown overnight in lysogeny broth (LB) (Sigma-Aldrich: Lennox, 10 g·L^–1^ tryptone, 5 g·L^–1^ yeast extract, 5 g·L^–1^ NaCl) at 37 °C and 130 rpm. *E. coli* AB1157 was only used for the propagation of the T4 phage, not as the host for any of the assays described below. The next morning, 1 mL of the overnight liquid cultures was used to inoculate 50 mL of fresh LB separately. This newly inoculated LB was incubated at 37 °C and 130 rpm until an OD_600_ (optical density at 600 nm) of 0.3 was reached. At this point, 100 μL of the bacteriophage stock was added to each corresponding flask and the samples were incubated for a further 4 h. The *E. coli* EV36 and AB1157 bacterial debris were pelleted by centrifugation at 3220 x*g* for 10 min before passing the supernatant through a 0.2 μm pore-size membrane filter. Two prepared phage stocks in LB were stored at 4 °C.

#### Cesium Chloride Purification of K1F-GFP and T4 Phages

For the purification of both bacteriophages (K1F and T4), the previously propagation assay described above was scaled up to 250 mL per sample by transferring the supernatant. Sodium chloride was added to each sample to achieve a final concentration of 1 M. After incubation on ice for 1 h, each phage sample was centrifuged at 3220 x*g* and the supernatant was filtered through at 0.2 μm pore-size membrane before adding PEG-8000 to a final concentration of 10% w/v. Both samples were left overnight at 4 °C, before centrifugation at 25 000 x*g* for 1 h. Phage pellets were resuspended in 6–7 mL of SM buffer I and passed through a 0.2 μm pore-size membrane, before undergoing concentration and purification in a CsCl gradient for 20 h at 150 000 x*g* and 4 °C. Following the centrifugation, the extracted phage band was first dialyzed in SM buffer I and twice dialyzed in SM buffer II to remove the CsCl. Purified phage samples were stored at 4 °C.

#### Cryopreservation

The purified bacteriophage samples were diluted to a final concentration of 1 × 10^7^ PFU·mL^–1^ in 500 μL of phage + additive aliquots. After placing the samples in either −20 or −80 °C freezers (the cooling rate was not recorded), the vials were left in the freezer for 13 days. After the cryopreservation, each sample was thawed to 20 °C on benchtops. For the freeze/thaw cycles, samples were frozen for 30 min before thawing.

#### Plaque Assay: Quantification of Bacteriophages

Bacteriophage titers for both K1F-GFP and T4 phages were determined via a soft agar plaque assay, using 0.7% agar top lysogeny broth agar (LBA).^61^ A 100 μL aliquot of the serially diluted cryopreserved phage was incubated with an equal volume of bacteria host cell lawn (∼1 × 10^8^ CFU·mL^–1^ (colony-forming units)) at room temperature for 15 min before the addition of 3 mL of liquid top agar (0.7% agar) and pouring over a solid 1.5% agar LBA plate. After an overnight (24 h) incubation at 37 °C, the individually distinct zones of clearance on plates (plaques) were enumerated and quantified as PFU·mL^–1^ (plaque-forming units) taking into account the serial dilution from frozen aliquots. The assays were carried out in triplicate, using duplicates for each biological repeat (*n* = 6).

#### Twenty-Four-Hour *E. coli* EV36 Growth Curves

Samples were grown in a FLUOstar Omega microplate reader at 37 °C taking measurements of the optical density (OD_600_ or Abs_600_) every 5 min over a 24 h period. The final concentration of the 1 × 10^6^ CFU·mL^–1^ bacteria host was added to each well of a 96-well plate and grown for 4 h at 37 °C with shaking to reach the log phase. During the log phase, the tested aliquots were added to each corresponding well of the plate including 1% v/v Chemgene surface disinfectant (a positive control) and bacteriophages with a final concentration of 1 × 10^6^ PFU·mL^–1^ with or without the polymer additives. All samples were grown shaking in lysogeny broth (LB) media in a total volume of 200 μL. Data was obtained using the MARS data analysis software (Version 5.10). The growth curve was carried out in triplicate, using technical duplicates for each biological repeat (*n* = 6).

#### Bacterial Viability/Bacteria Eradication Assay: Quantifying of *E. coli* Colonies at Various Time Points

At three time points of interest, 7 h (the first dip in the growth curve), 10 h (slowing of the *E. coli* repopulation rate), and 24 h (end of OD readings), the CFU·mL^–1^ of the *E. coli* was determined using a modification of the previously described plaque assay, termed “viability assay”. Aliquots of 100 μL of serially diluted bacteria/K1F-GFP phage extractions taken directly from corresponding wells of the 96-well plate in the plate reader with final volumes varied according to time points were spotted and spread over a 1.5% agar LBA plate. After an overnight (24 h) incubation of the plates at 37 °C, the number of *E. coli* colonies were counted to determine the CFU·mL^–1^, accounting for sample dilutions. A FLUOstar Omega microplate reader was used and data was obtained from the MARS data analysis software. Each assay was carried out in triplicate, using technical duplicates for each biological repeat (*n* = 6).

## Results and Discussion

**Figure 1:**
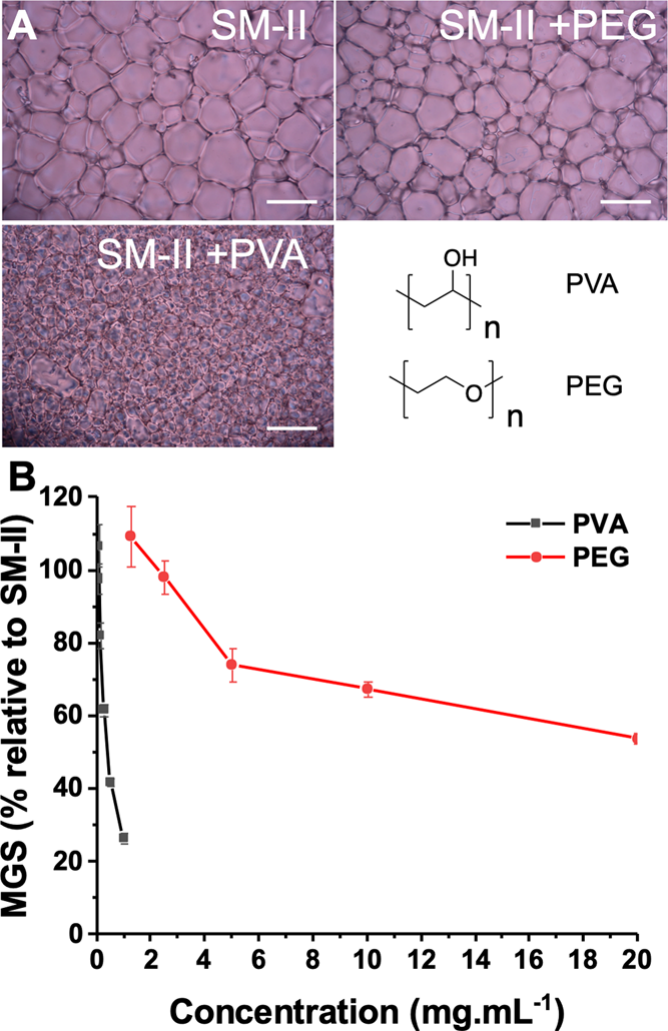
Ice recrystallization inhibition activity of polymers in SM-II buffer. (A) Example micrographs of ice wafers after 30 min annealing at −8 °C. PEG (4000 g·mol^–1^), 10 mg·mL^–1^, and PVA (10 000 g·mol^–1^), 1 mg·mL^–1^. Scale bars = 100 μM. (B) IRI activity as a function of polymer concentration. MGS = mean grain size relative to SM-II control.

Our initial hypothesis was that addition of ice recrystallization inhibiting (IRI) polymers may mitigate cold-induced damage to the phage, in particular by reducing the stress during the thawing stage. The IRI-active polymer PVA (poly(vinyl alcohol)) has shown benefit for protein storage^57^ (by reducing aggregation), as well as bacteria,^62^ hence was chosen due to its ease of use and commercial availability. To ensure PVA retained IRI activity in the buffer used (SM-II) for handling the phage, the “splat” assay was used to evaluate ice growth.^59,63^ In this assay, small ice crystals were nucleated and then allowed to grow at a subzero temperature (−8 °C) and their mean grain size (MGS) is reported relative to the buffer/media alone. This test is crucial, as solvent conditions can enhance/reduce the IRI activity^64^ and saline is essential to avoid false positives.^59^Figure 1 shows the structures of the polymers tested (PVA and PEG), for example, ice crystal wafers and the MGS activity. As expected, PVA retained its IRI activity inhibiting all growth at 1 mg·mL^–1^ but was slightly less active than what is seen in standard-phosphate-buffered saline (PBS) buffer, attributable to the additional solution components. PEG shows no significant IRI activity in the concentration range tested (noting that IRI is a continuum not on/off property and very high concentrations of any polymer will slow growth^63^). With this to hand, the polymers could be used for phage testing, as shown below.

**Figure 2:**
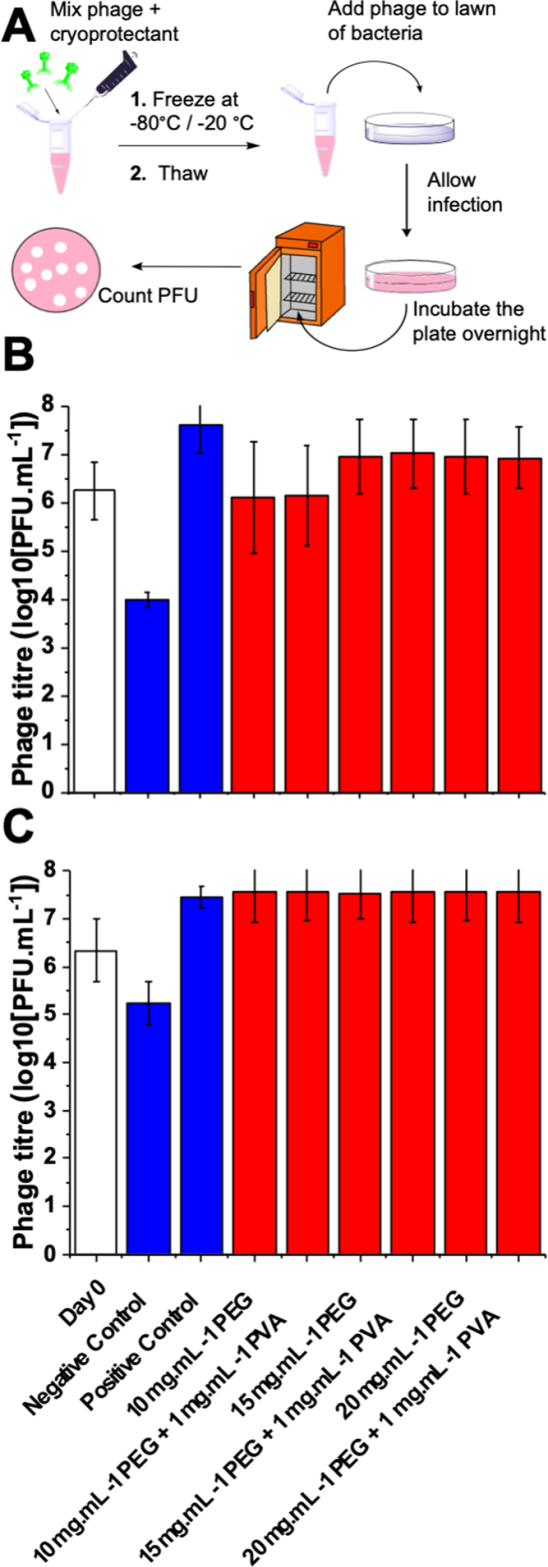
K1F-GFP phage cryopreservation and recovery with a single freeze (−80 °C)/thaw (20 °C) cycle. (A) Schematic of freeze/thaw and quantification of phage recovery. (B) Phage titer after cryopreservation at −20 °C. (C) Phage titer after cryopreservation at −80 °C. Each sample was 500 μL. Errors bars are standard deviations from three biological replicates and two technical replicates.

K1F was chosen as a model phage to check different synthetic cryoprotectants. To evaluate phage recovery, the plaque-forming units of infectious phage particles were quantified after the thaw. This was achieved by inoculating the *E. coli* host, EV36,^65^ and measuring the phage titer by the number of plaques formed, following a standard bacterial infection procedure called a plaque assay (see the Experimental Section). The EV36 strain of *E. coli* is a K12/K1 hybrid, meaning that it is a nonpathogenic lab strain that expresses the K1 capsule.^66^ The K1 capsule is associated with pathogenicity.^66^ Thus, this strain is used as a model for pathogenic *E. coli* but without the hazards associated with working with a strain expressing other pathogenic genes. K1F is a T7-like phage but has an endosialidase gene in the position of the usual T7 tail fiber gene.^67^ This means that, unlike T7, K1F has the ability to break down the K1 capsule and making it of interest as a clinical treatment.^68^ To evaluate cryopreservation conditions, the K1F-GFP phage (∼1 × 10^6^ PFU·mL^–1^) (plaque-forming units) was mixed with the cryopreservation solutions, frozen to either −20 or −80 °C (representing a standard and ultra-low-temperature freezer) for 13 days. After the thaw, the diluted lytic phage was grown on a lawn of its host bacteria, and by counting zones of clearance, the PFUs (plaque-forming units) were quantified (Figure 2A). The positive control for this study was 50 wt % glycerol (a commonly used reagent for phage cryopreservation).^69^ Recovery data is shown in Figure 2B/C. The total phage recovered shown by the positive control cryoprotected sample is visibly higher at −20 °C compared to −80 °C, which is partly due to the fact that 50 wt % glycerol does not freeze in a standard freezer, resulting in a chilled state of the aliquot for the 13 days period, avoiding any freeze–thaw damage. After storage at −20 °C, it was clear that all solutions containing PEG showed higher recovery, up to 100-fold, than SM-II buffer alone (negative control), as shown in Figure 2B. Our original hypothesis was that PVA (as an IRI-active component) would enhance post-thaw yields, but in each case, there was no change in the phage titer compared to the formulations with no PVA. PVA alone (Supporting Information, Figure S1) also showed no benefit. Changing to −80 °C cryopreservation, all polymer formulations matched the performance of 50 wt % glycerol (Figure 2B). This was a surprising level of recovery, considering the polymers were used at a 50-fold lower concentration than glycerol. As seen after storage at −20 °C, there was no impact of the PVA on phage recovery after storage at −80 °C when compared to the negative control (Supporting Information, Figure S1).

These initial results confirmed that a macromolecular cryoprotectant for phage cryopreservation is not only feasible but very potent. It also showed that (under the present conditions, noting that volume and thawing rates can all play a role) ice recrystallization is not a major stress factor for phage cryopreservation when performed in the presence of PEG. For solvent-free bacteria cryopreservation using IRI-active materials, it has been observed that an additional hydrophilic polymer (such as PEG) was essential for the IRI-active polymer to provide benefit and hence a similar effect may be occurring here. PEG is also known to stabilize proteins during freeze–thawing,^70,71^ via a proposed preferential steric exclusion of PEG from the surface of proteins, alleviating any potential deactivation, in addition to their hydration.^72−74^ Previous studies^69^ have shown that concentrations of PEG at both 10 and 45% w/v led to similar mean survival times of rabies virus compared to similar concentrations of other cryoprotectants, sucrose, DMSO, and glycerol, after 30-day storage at −20 °C. A discussion on the potential role of osmotic stress is included later in this manuscript.

**Figure 3:**
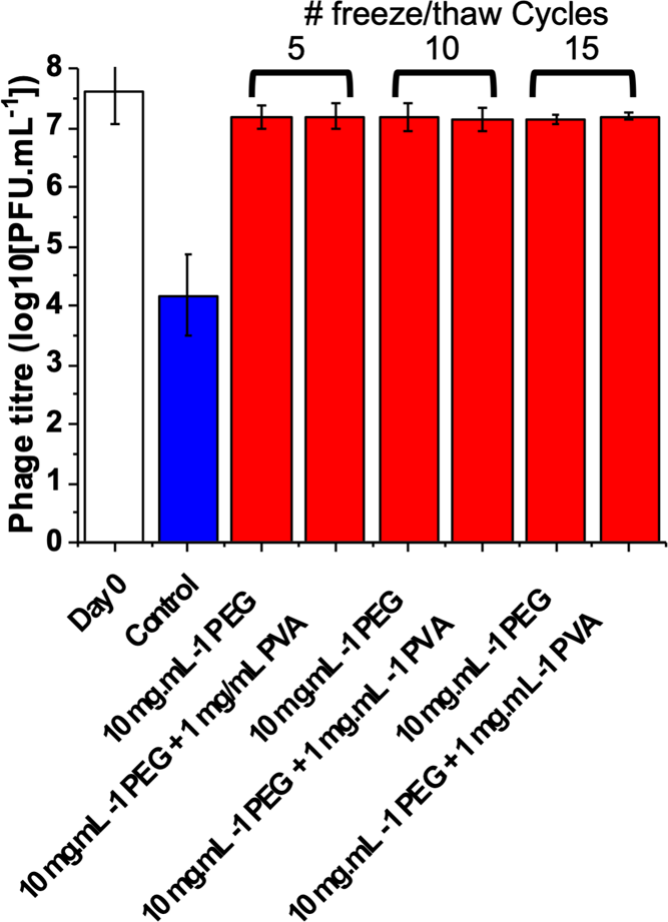
Post-thaw recovery of the K1F-GFP phage following variable freeze (−80 °C)/thaw (20 °C) cycles. Control is PVA [1 mg·mL^–1^] after 15 cycles. Error bars represent SD from three biological and two technical replicates.

The above data was from a single freeze–thaw cycle. Therefore, as a more robust challenge, the phage was exposed to a series of 5, 10, or 15 freeze (−80 °C)/thaw (20 °C) cycles. Repeated freeze–thaw cycles may cause extended freeze–thaw damage, through deliberate or accidental warming of samples. PVA was included again, to ensure that any excess ice recrystallization damage could be probed (Figure 3). In each case, nearly full recovery of the phage, compared to the day 0 control, was achieved with just 10 mg·mL^–1^ PEG. The PVA again showed no significant impact (neither positive nor negative). This data showed that the polymeric cryoprotectant strategy is suitable for repeated use, for e.g., a research environment, where stocks may be thawed, sampled, and refrozen, with no detriment to the sample function and integrity.

**Figure 4:**
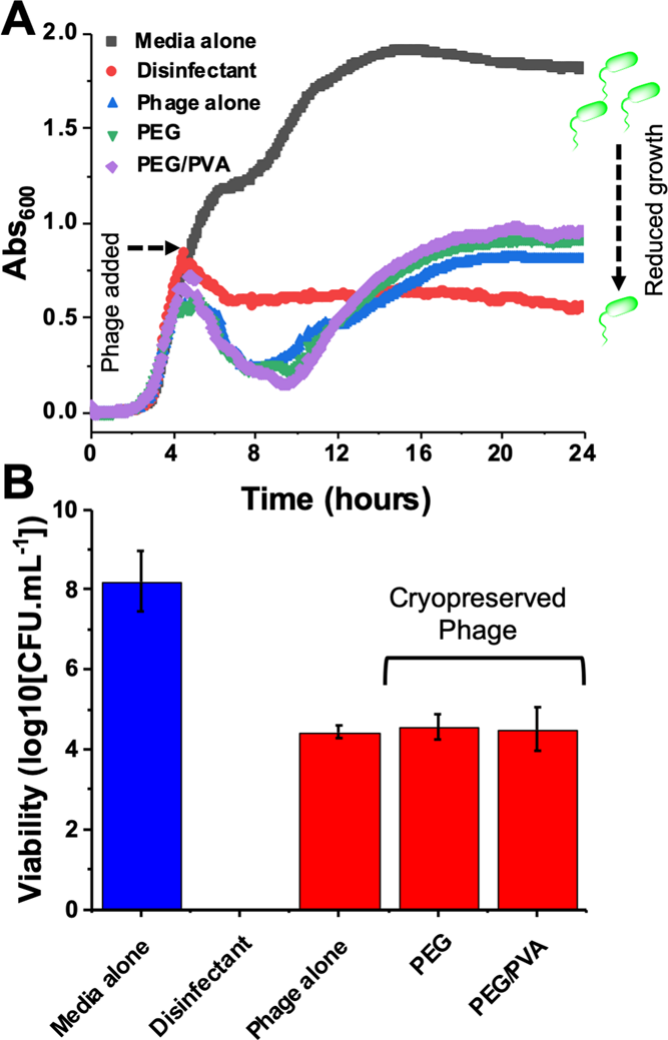
*E. coli* growth inhibition by the K1F-GPF phage. (A) Growth curves of *E. coli* EV36 upon addition of the phage or controls. Phages were added during the log phase (4 h) to a final concentration of 1 × 10^6^ PFU·mL^–1^. PEG and PEG/PVA refer to the cryopreserved phage using those additives. The K1F-GFP phage was freeze (−80 °C)–thawed (20 °C); (B) *E. coli* EV36 viability at 7 h, determined by extraction of the sample from the growth experiment (part A) and plating and counting colony-forming units. [Disinfectant] = 1% v/v Chemgene; [PEG] = 10 mg·mL^–1^; [PEG/PVA] = 10 + 1 mg·mL^–1^. Error represents SD from three biological and two technical replicates.

The above data shows that the addition of polymers as cryoprotectants allows increased post-thaw yield of the K1F-GFP phage. One intended application of phage is a therapy to kill pathogenic bacteria, as an alternative to traditional antibiotic treatments, or as a phage-antibiotic synergy (PAS) aiming to reduce the dose of antibiotics and the development of antimicrobial resistance (AMR).^75^ The maintenance of efficacy and the lytic ability of our phage after cryopreservation was checked by measuring changes in optical density (OD_600_) in a growth curve over a 24 h period. In this assay, the phage was cryopreserved with 10 mg·mL^–1^ PEG, thawed, and then added to a culture of *E. coli*, and the change in turbidity was measured by absorbance at 600 nm. In media alone, the *E. coli* reached a plateau (stationary phase) within 12 h and a positive control of 1% v/v Chemgene HLD4L disinfectant (containing didecyldimethylammonium chloride) arrested all growth in the same time period. Addition of the noncryopreserved phage successfully prevented bacterial growth up to 8 h, after which time growth recovered (as the dose did not irradicate all *E. coli* and there was resistant strain outgrowth). The phage cryopreserved with both PEG or PEG/PVA showed similar performance in this assay. Bacteria were also quantified at the 7 h time point, via cell plating and quantifying the number of colony-forming units (Figure 4B). Additional plaque-forming units (PFU) and colony-forming units (CFU) measured at the 7 h, 10 h, and 24 h time points are included in the Supplementary Information (Figures S2 and S3). The disinfectant (positive) control showed no live bacteria (limit of detection = 1 log CFU·mL ^–1^). After treatment with the cryopreserved phage, statistically similar bacterial titers were measured when compared with treatment with the fresh phage samples. This data shows that the cryopreserved phage, using biocompatible polymers, could potentially be used as frozen phage stocks ready to be deployed, by simple thawing, for use in emerging therapeutic applications, with their performance matching that of the fresh phage.

While the genetic and molecular approaches to understanding the growth, metabolism, adaptability, and physiology of bacteria (such as our *E. coli* EV36 host) have focused on studying planktonic cells in batch cultures, many bacteria (*E. coli* included) live primarily in immobile communities, referred to as biofilms.^76^ Besides being the major cause for recurrent urinary tract infections (UTI), *E. coli* biofilms are one of the pathogens commonly responsible for medical device-related infectivity.^77^ Therefore, as a second method of comparing the performance of fresh and cryopreserved K1F-GFP bacteriophages, *E. coli* biofilm eradication was investigated (Supporting Information, Figure S4). The cryopreserved phage showed similar effects as the fresh phage (∼ 10-fold reduction in bacterial CFU) after a single application. However, compared to the positive control (1% v/v Chemgene) despite the slight reduction in the *E. coli* CFU, the phages (both cryopreserved as fresh) were inefficient at eradicating the mature biofilm (72 h grown). As 24 and 48 h grown biofilms were not investigated, the thickness of the biofilm cannot be ruled out as a contributing factor to phage eradication inefficiency.

**Figure 5:**
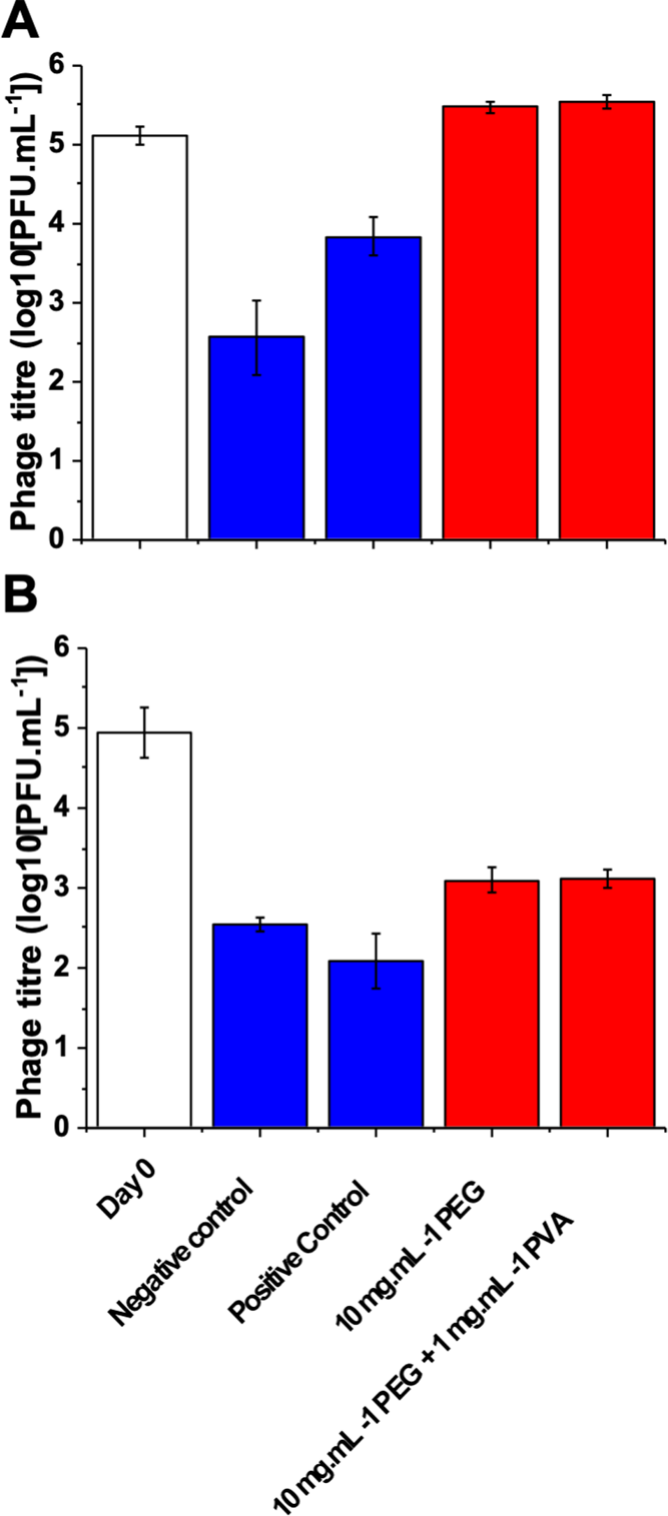
T4 phage titers after one freeze–thaw cycle at −20 °C (A) and −80 °C (B) freezing for 13 days. Day 0 = titer on the day of freezing; negative control = the phage without the additive at −20 and −80 °C; positive control = 50 wt %. glycerol; red = phage and additives. Cryopreserved samples were 500 μL. Error represents standard deviation from three biological and two technical replicates.

The above data was using a K1F-GFP phage, so it was important to evaluate if other phages responded in a similar manner. The T4 bacteriophage was chosen due to being a well-studied model phage for *E. coli.*(78) Like K1F, T4 encodes its own replication proteins, bypassing the host replication machinery.^79^ T4 is also clinically relevant, with evidence of low immunogenicity in oral application and with potential uses for oral vaccine development.^80^ Due to its clinical relevance and the breadth of information already available on T4, it is an ideal candidate to evaluate our cryoprotectants. Identical freeze–thaw conditions were applied, as used previously, and the results of the cryopreservation at both −20 and −80 °C are shown in Figure 5. It was observed that the T4 phage was more susceptible to cold damage than the previously used K1F and that 50% glycerol provided little protection at both freezing temperatures. It should be noted that the EV36 strain is not the ideal host for this T4 phage, as the K1 capsule blocks the phage receptors. However, EV36 is a hybrid of K1/K12, so infection with the T4 phage (which naturally targets K12) still leads to plaque formation, which was visibly smaller in size compared to K1F plaques, as EV36 is not the natural host for T4. The same *E. coli* EV36 host was used here though, to allow comparison in the present context. As seen for the K1F phage, addition of the polymeric cryopreservation formulation in all cases leads to a greater post-thaw phage titer of approximately 100-fold. This increase, if put in the context of therapy, would mean that using this polymeric cryopreservation strategy would deliver a 100-fold higher dosage compared to a glycerol frozen solution or allow more treatments from a single stock. The utility across two distinct classes of phages also suggests that this approach could be used to generate bespoke phage cocktails, although this will be the subject of a future investigation.

As further controls, we tested poly(vinylpyrrolidone) and hydroxyethyl starch for phage cryopreservation, as both of these polymers are widely used in formulations or for cryopreservation.^81,82^ In the initial screening (Supporting information, Figure S5), both PVP and HES increased post-thaw titers of the K1F phage, to an extent less than PEG. However, based on our preliminary results, we cannot conclude which one is more efficient and a full comparison of the materials would be required. To the best of our knowledge, there are no studies on the use of polymer-only solutions to preserve the bacteriophage, but our data suggests that hydrophilic polymers can be used as a simplistic cryoprotectant solution and a detailed structure–function study of this will be undertaken in the future, as well as the response of different phage strains. There is evidence that osmotic stress plays a key role in phage storage. As shown by Duyvejonck,^83^ several myoviridae and podoviridae (groups encompassing T4 and T7 phages, respectively) were resistant to storage conditions in Dulbecco’s phosphate-buffered saline (DPBS) (with and without Ca^2+^/Mg^2+^) with maintained infectivity up to 554 and 243 days, respectively, at 4 °C. Interestingly, when the T4B phage was rapidly transferred from a concentrated to a dilute solution, retained phage activity depended on the initial salt concentration of the solution in which it was suspended.^84^ Phage inactivation observed during rapid dilution did not occur during slow dilutions. A rapid change in osmotic pressure caused by the change in salt concentration may cause the phage DNA to extrude from the tail or their heads to break, as observed by Lark and Adams.^85^ In our study, the addition of the PEG may affect the water diffusion rates in the unfrozen channels formed during freezing, due to increased viscosity, which has been seen for glycerol (positive control used here)^86^ and mitigated the osmotic stress encountered. High concentrations of PEG (10–45 wt %) have been shown to protect against rabies virus during cryopreservation.^69^

## Conclusions

Here, we report that the bacteriophage can be successfully cryopreserved across a range of temperatures by the addition of poly(ethylene glycol), PEG, and other hydrophilic polymers, as an alternative to some of the currently used buffers, such as those containing glycerol. K1F and T4 bacteriophages were used to evaluate performance. It was found that just 10 mg·mL^–1^ (∼1 wt %) of PEG allowed plaque-forming unit recovery matching that from a positive control of 50 wt % glycerol at −80 °C but slightly underperforming at −20 °C. It should be noted that the glycerol solutions do not actually freeze at −20 °C, making direct comparisons challenging but showing that conventional laboratory freezers can be used to freeze the phage using our polymeric system. Using the polymer formulation, near 100% phage recovery was achieved (represented as plaque-forming units), even after 15 freeze/thaw cycles, demonstrating that this provides robust protection, which can be applied to larger sample sizes in a practical setting. The role of ice recrystallization was probed using the potent ice recrystallization inhibitor poly(vinyl alcohol), PVA, which has been found to be useful in other cryopreservation scenarios. In all cases, addition of PVA showed no significant benefit, suggesting that ice growth during thawing and hence irreversible aggregation are not a major mechanism of damage for the phage. The polymers may be reducing osmotic stress, by impacting diffusion in unfrozen channels, but more research is needed to elucidate a mechanism. In *E. coli* kill studies, the cryopreserved phages were found to match the performance of fresh phages, demonstrating that this approach may allow for frozen pure or cocktails of the phage, intended for therapy, to be stored as cryopreserved stocks. As PEG is biocompatible, has low immunogenicity, and is edible, it would not need to be removed after the thaw. Preliminary data also showed that other hydrophilic polymers can provide this protection and that the protective capacity is not unique to PEG and that protecting the phage from cold damage (compared to other biologics) may be relatively straightforward. The extent and magnitude of protection between different macromolecular chemistries and architectures will form the basis of future studies.

## Supporting Information Available

Additional post-thaw recovery data and biofilm disruption data and methods are included (PDF)

## Notes

Background data is available at wrap.warwick.ac.uk
