# Supplementary material for: Polymer-Mediated Cryopreservation of Bacteriophages
Source: Biomacromolecules. 2021 Nov 30;22(12):5281–9. doi: 10.1021/acs.biomac.1c01187 (PMC8672357; doi:10.1021/acs.biomac.1c01187)
Supplement: Supplementary file 1 — bm1c01187_si_001.pdf [file bm1c01187_si_001.pdf]

Supporting Information for:

Polymer-Mediated Cryopreservation of Phage

Huba L. Marton,[a] †Kathryn M. Styles,[c]† Peter Kilbride,[d] Antonia P. Sagona[c]* and Matthew I. Gibson[a,b,*]

[a)] Department of Chemistry, University of Warwick, Coventry, CV4 7AL, U.K.

[b)] Warwick Medical School, University of Warwick, Coventry, CV4 7AL, U.K.

[c)] School of Life Sciences, University of Warwick, Coventry, CV4 7AL, U.K.

d) Asymptote, Cytiva, Chivers Way, Cambridge, CB24 9BZ, U.K.

†Both authors contributed equally

CORRESPONDING AUTHOR DETAILS

*Fax: +44 247 652 4112. E-mail: m.i.gibson@warwick.ac.uk and A.Sagona@warwick.ac.uk

Additional Data.

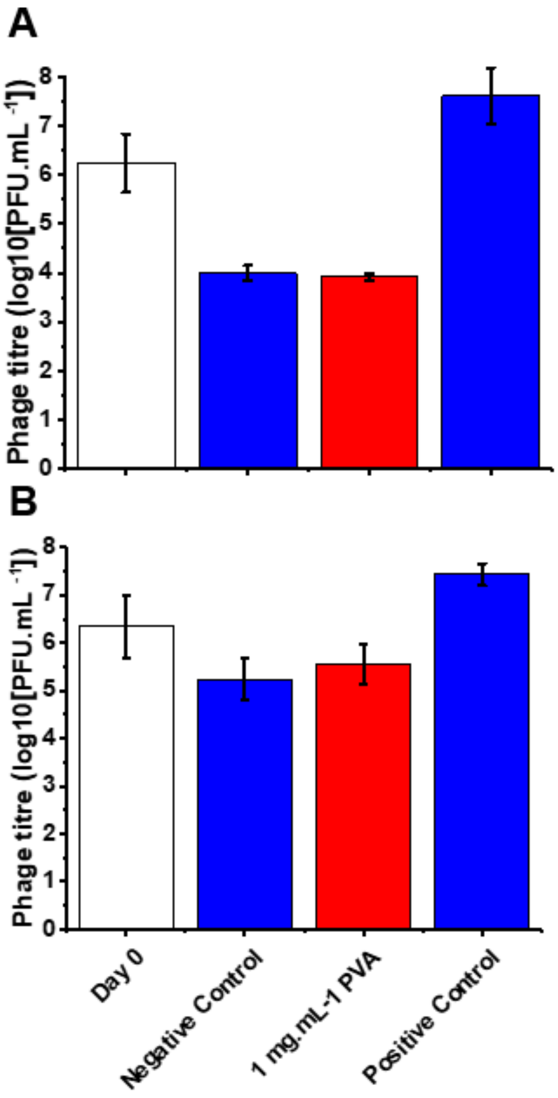

Figure S1. K1F- GFP phage cryopreservation and recovery with single freeze (-80 °C)/thaw (20 °C) cycle. A) Phage titre after cryopreservation at -20 °C; B) Phage titre post-cryopreservation at – 80 °C. Each sample was 500 µL. Errors bars are standard deviations from three biological and two technical replicates.

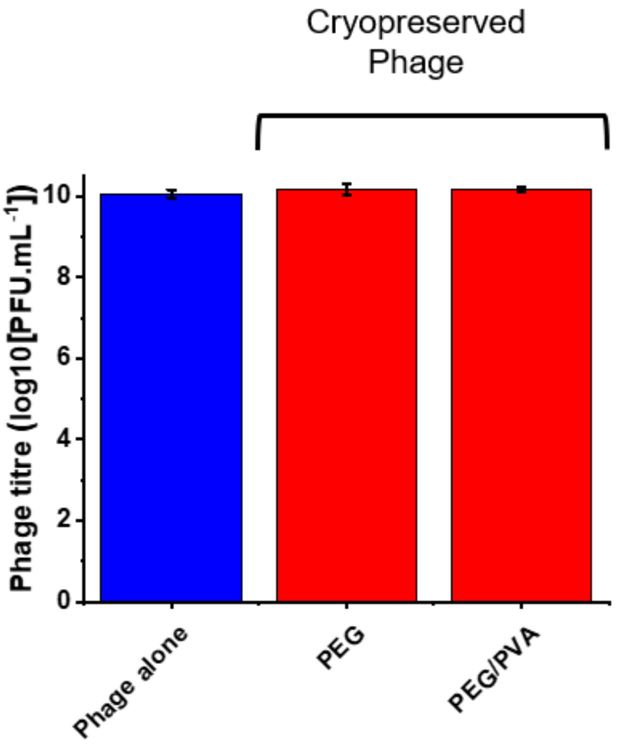

Figure S2. K1F-GFP bacteriophage phage titre at 7 h, determined by extraction of sample from growth experiment (Figure 4A), plating and counting plaque forming units. [Phage alone] = fresh phage without additive; [PEG] = 10 mg.mL^{-1}; [PEG/PVA] = 10 + 1 mg.mL^{-1}. Error represents SD from three biological and two technical repeats.

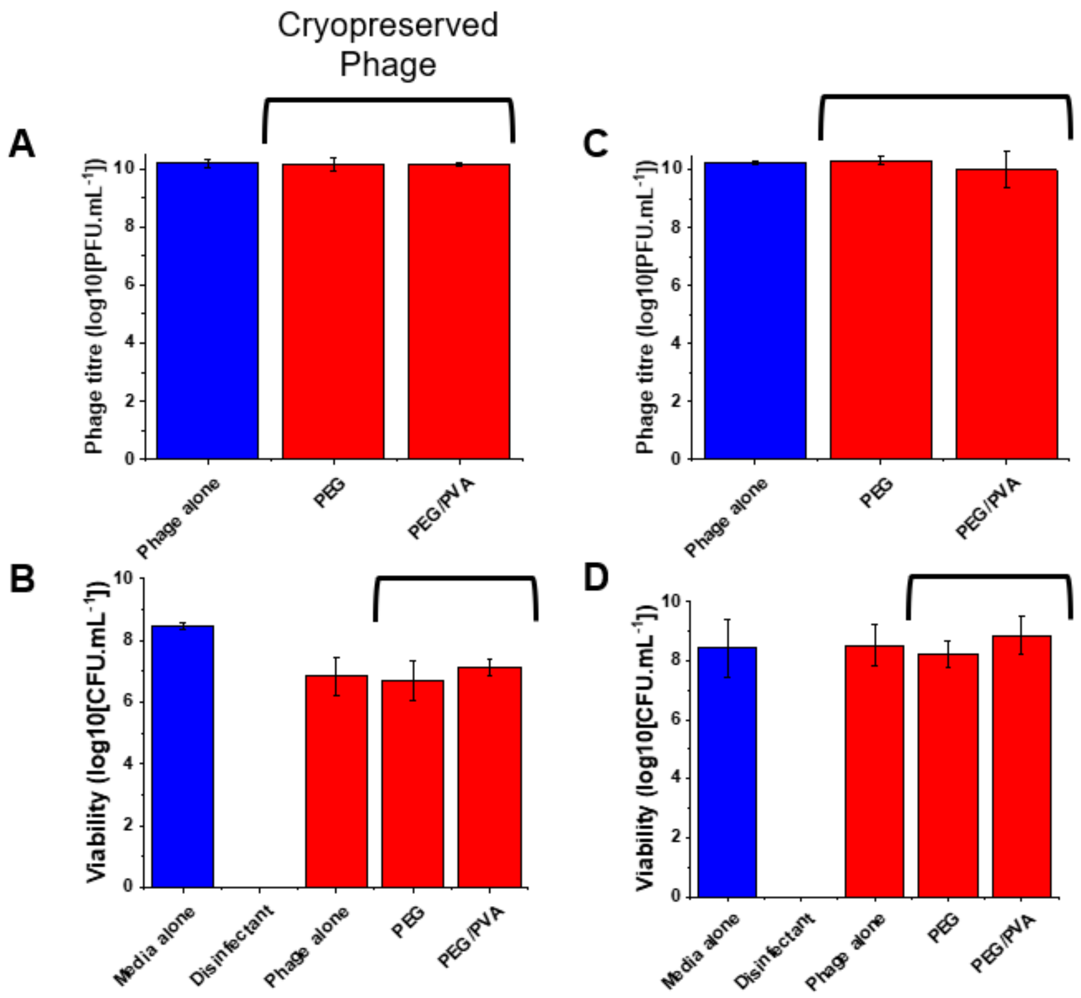

Figure S3. *E. coli* growth inhibition by K1F-GPF bacteriophage. A) and C) K1F-GFP bacteriophage phage titre at 10 and 24 h, determined by extraction of sample from growth experiment (Figure 4A), plating and counting plaque forming units. B) and D) *E. coli* EV36 viability at 10 and 24 h, obtained by extraction of sample from growth experiment (Figure 4A), plating and counting colony forming units. [Phage alone] = fresh phage without additive, cryopreserved phage marked with bracket; [PEG] = 10 mg.mL^{-1} ; [PEG/PVA] = 10 + 1 mg.mL^{-1}. Error represents SD from three biological and two technical repeats.

E. coli EV36 biofilm eradication assays.

From a freshly grown *E. coli* EV36 culture plate, an inoculum was prepared to an OD_{600} of 0.1 (equivalent to ~1 x 10^8 $CFU.mL^{-1}$), which was serially diluted to achieve a suspension with a final concentration of 1 x 10^5 $CFU.mL^{-1}$. 150 µL of the suspension were added to the wells of a 96-well plate, covered using a lid with pegs and incubated at 37 °C and 110 rpm for 72 h in a damp environment in the FLUOstar Omega microplate reader. At that point, the mature biofilms (72 h grown) were washed three times with 200 µL PBS to remove planktonic bacteria. After the final wash, the *E. coli* biofilm pegs were transferred to plates, containing K1F-GFP bacteriophages with a final concentration of 1 x 10^6 $PFU.mL^{-1}$ or the positive control of 1% v/v Chemgene separately. These plates were then incubated statically at 37 °C for 24 h. Post incubation, biofilms from the pegs were transferred to fresh PBS recovery plates by sonicating in a sweeping water bath for 5 min. Viability assays (described above) were used to quantify the colony forming units for the *E. coli* EV36 biofilms and plaque assays (described above) were used to quantify the corresponding plaque forming units of the K1F-GFP bacteriophage used for each sample tested. Each assay was carried out in triplicate, using technical duplicates for each biological repeat (n=6).

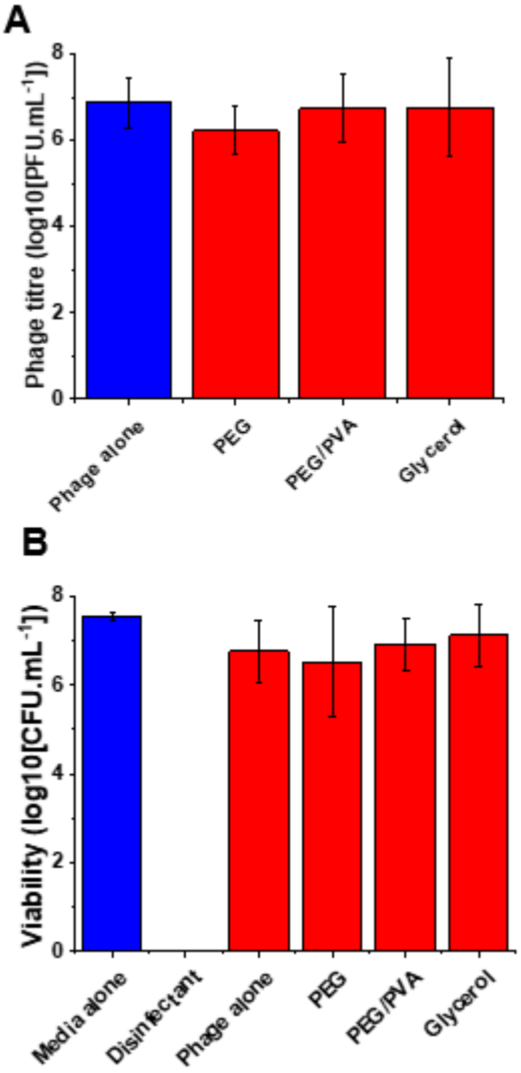

Figure S4. *E. coli* biofilm eradication by K1F-GPF bacteriophage. A) Phage titre of K1F after 24 h incubation in mature (72 h grown) biofilms quoted as plaque forming units. B) *E. coli* EV36 biofilm viability post 24 h phage treatment presented as colony forming units. [Phage alone] = fresh phage without additive; [Disinfectant] = 1% v/v Chemgene; [PEG] = 10 $mg.mL^{-1}$; [PEG/PVA] = 10 + 1 $mg.mL^{-1}$; [Glycerol] = 10 $mg.mL^{-1}$. Error represents standard deviations from three biological and two technical repeats.

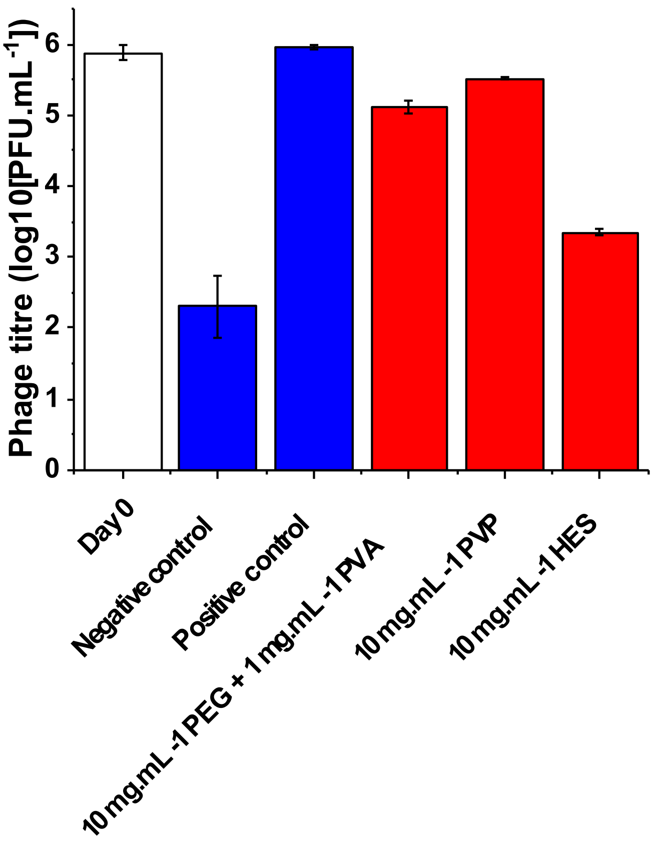

Figure S5. K1F-GFP bacteriophage cryopreservation and recovery with a single freeze (13 days) (-80 °C) /thaw (20 °C) cycle. Day 0 = titre on day of freezing (white); negative control = phage without additive; positive control = 50 % wt. glycerol; red = phage and additives. Cryopreserved samples were 500 μL. Error represents standard deviation from two technical repeats (1 biological).
